# Automated data abstraction for quality surveillance and outcome assessment in radiation oncology

**DOI:** 10.1002/acm2.13308

**Published:** 2021-06-08

**Authors:** Rishabh Kapoor, William C. Sleeman, Joseph J. Nalluri, Paul Turner, Priyankar Bose, Andrii Cherevko, Sriram Srinivasan, Khajamoinuddin Syed, Preetam Ghosh, Michael Hagan, Jatinder R. Palta

**Affiliations:** ^1^ Department of Radiation Oncology Virginia Commonwealth University Richmond VA USA; ^2^ National Radiation Oncology Program US Veterans Healthcare Administration Richmond VA USA; ^3^ Department of Computer Science Virginia Commonwealth University Richmond VA USA

**Keywords:** big data in radiation oncology, quality surveillance

## Abstract

Rigorous radiotherapy quality surveillance and comprehensive outcome assessment require electronic capture and automatic abstraction of clinical, radiation treatment planning, and delivery data. We present the design and implementation framework of an integrated data abstraction, aggregation, and storage, curation, and analytics software: the Health Information Gateway and Exchange (HINGE), which collates data for cancer patients receiving radiotherapy. The HINGE software abstracts structured DICOM‐RT data from the treatment planning system (TPS), treatment data from the treatment management system (TMS), and clinical data from the electronic health records (EHRs). HINGE software has disease site‐specific “Smart” templates that facilitate the entry of relevant clinical information by physicians and clinical staff in a discrete manner as part of the routine clinical documentation. Radiotherapy data abstracted from these disparate sources and the smart templates are processed for quality and outcome assessment. The predictive data analyses are done on using well‐defined clinical and dosimetry quality measures defined by disease site experts in radiation oncology. HINGE application software connects seamlessly to the local IT/medical infrastructure via interfaces and cloud services and performs data extraction and aggregation functions without human intervention. It provides tools to assess variations in radiation oncology practices and outcomes and determines gaps in radiotherapy quality delivered by each provider.

## 1 INTRODUCTION

Advanced technologies in health care are bringing a sharper focus on clinical outcome assessment and the assessment of health care quality. Manual abstraction, collation, and subsequent analysis of health care quality from patient treatment and outcome data are onerous, expensive, and impractical. Advances in computer storage, computing power, and the ability to electronically mine data from disparate sources (e.g., demographics, genetics, imaging, treatment, clinical decisions, and outcomes) have enabled big data research in medicine. The evolution of several initiatives in the realm of interconnectivity of health care data sources and the availability of advanced computing frameworks have opened doors for answering a broad array of questions related to quality, safety, and outcomes of patients’ clinical care efficiently, objectively, and in a cost‐effective manner.

**Fig. 1:**
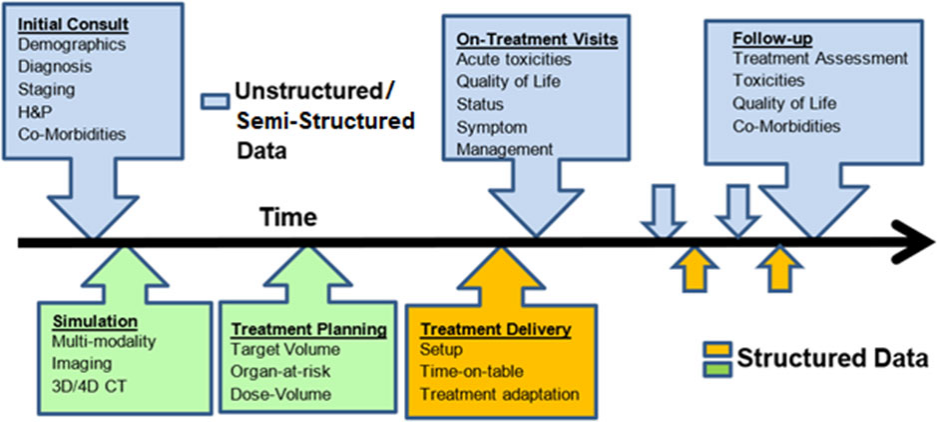
The sequential radiation treatment workflow: initial patient consultation, simulation, treatment planning, treatment delivery, on‐treatment evaluation, and follow up. The clinical data are in unstructured and/or semi‐structured data formats, whereas simulation treatment planning and treatment delivery data are inherently in a structured format.

In the radiation oncology domain, large amounts of data are captured routinely across several clinical systems over the course of a patient’s treatment as shown in Fig. 1.

The electronic health record (EHR) is used to document clinical data that typically include demographic information, medical history, medications, laboratory test results, and radiology reports. The physician assessments are often stored in unstructured free text from which key data elements are difficult to abstract for any subsequent data mining efforts. For each patient receiving radiotherapy treatment, the clinical documentation in EHR typically includes (a) a detailed initial consultation note, (b) a simulation note describing the treatment simulation procedure, (c) a treatment planning note documenting the prescription and proposed treatment plan, (d) a weekly on‐treatment visit (OTV) note from the staff physician documenting a review of the patient’s treatment progress and any acute side effects, (e) a treatment summary or survivorship care plan for the patient and referring physician at the completion of therapy, and (f) routine follow‐up notes tracking disease outcomes and any late toxicities. These clinical notes are usually dictated on a telephone, transcribed, and imported into the EHR as preliminary documents. These free‐text formatted notes are then reviewed, edited, and finalized. There is a wealth of information in clinical notes for big data applications, but the challenge is to capture and abstract these data in discrete format as part of the regular clinical workflow. However, the treatment planning data including the radiotherapy plan, images, dose, structure set, and dose‐volume information from the treatment planning system (TPS) are in structured formats (DICOM‐RT). Additionally, the treatment management system (TMS) that contains information regarding the radiation treatment delivery, fractions, visits, and so on is also structured.

The challenge in radiation oncology is to aggregate data, which are both structured and unstructured from disparate data sources. It is extremely difficult to clean, parse, and collate the data intelligibly, thus making many research and operational tasks that deal with the optimization of quality care, research‐based analysis of radiation treatment, and diagnosis‐based research and development of computer‐aided diagnostic tools at the infrastructural level quite difficult. Additionally, the lack of interconnectivity and interoperability of RT software systems has made the process of data sharing/transfer cumbersome and challenging. Unfortunately, valuable clinical and radiation treatment data remain trapped behind proprietary software systems. There are natural language processing (NLP) methods that can be employed to extract structured data from clinical narratives dictated in the EHR. These methods utilize text mining symbolic methods approaches such as named entity recognition (NER) based on dictionary lookup and information extracting (IE) relying on pattern matching. Each of these methods provides far from ideal results in gathering accurate structured information from the clinical notes because these methods have to deal with the idiosyncrasies of clinical sublanguage due to the use of nonstandard ontologies and data dictionaries as well a high degree of spelling and grammatical errors.^1^ The accuracy of these approaches can potentially improve when there is a comprehensive cancer ontology used to enable semantic representation of textual information founds in clinical narratives. The utilization of these not perfect methods for extracting structure data from the EHR can adversely affect the outcome assessment and predictive analytics modules specifically considering the sensitivity of the data elements to both tasks. Therefore, the structured template‐based approach alleviates these concerns and makes structured data capture more credible for clinical use in production quality assurance (QA), outcomes, and big‐data analytics platforms.

The Veteran Health Administration’s National Radiation Oncology Program (NROP) office embarked on an initiative to develop an integrated enterprise‐wide data curation, storage, and analytics portal, called Health Information Gateway and Exchange (HINGE). HINGE is a web‐based electronic structured data capture system that has electronic data sharing interfaces with the EHR, TMS, and TPS with a specific goal to collect accurate and comprehensive data and to determine clinical practice variations, outcomes, and gaps in treatment quality and to compare the effectiveness of various treatment modalities and ultimately enable *big data* analytics in radiation oncology. It is an automatic data aggregator that collates data from different radiotherapy clinical systems/IT applications. It processes the treatment data for quality assessment, predictive analytics, and other enterprise‐driven clinical informatics solutions with a single online data portal and provides benchmark data and quality improvement tools for individual providers. Additionally, HINGE’s design and infrastructure caters to the imminent need for a research‐based practice environment and is cognizant of the role of advanced modern computational strategies involving big‐data predictive analytics and clinical informatics. Because we realized that achieving these objectives for the whole cancer domain would be extremely challenging, we restricted our scope to two disease sites (prostate and lung cancer). The promise does not come without challenges, and hence, there were significant technical and workflow‐related challenges with the actual extraction and aggregation of data from disparate radiotherapy information sources.

## 2 IMPETUS FOR AUTOMATED RADIOTHERAPY DATA ABSTRACTION

The Veterans Health Administration (VHA), which is the largest integrated health care system in the United States, provides care at 1243 health care facilities, including 170 VA medical centers and 1063 outpatient sites of care of varying complexity. It serves more than 9 million enrolled veterans each year. Forty of the large VA medical centers offer onsite radiation oncology services with the oversight from NROP office. In 2016, the NROP office embarked on a pilot project to monitor the quality of radiotherapy delivered, determine practice variations, and identification of the care gaps in the VHA. The pilot effort addressed intermediate risk and high‐risk prostate cancers (CaP), stage IIIA/B nonsmall cell lung cancers (NSCLCs), and limited stage small cell lung cancers (SCLCs). These disease site presentations were selected for the pilot because radiotherapy (RT) pivotal in the treatment of these cancers, which together represent more than 60% of patients receiving RT in the VA.

The VHA NROP office collaborated with the American Society for Radiation Oncology (ASTRO) to establish clinical quality measures (CQMs) by which individual patient care would be assessed and compared with the national VHA practice. ASTRO assembled disease site panels composed of nationally recognized experts who were asked to identify CQM for each phase of patient management by the radiation oncologist as well as dose/volume metrics for the evaluation of quality of radiation treatment plans. The genesis of CQM was the seminal body of work done by the American College of Radiology’s Quality Research in Radiation Oncology program.^2^, ^3^ Panels defined CQM in three categories: currently expected performance measures, those anticipated for the near future (aspirational CQM), or CQM for surveillance only. Methods were developed for manual data abstraction, analytic methods for DICOM‐RT data, data curation, and the data scoring system. Web‐based user interfaces were also developed to report patient scores to their VHA radiation oncologists and aggregate data for benchmarking. Data elements for 1567 patients from the 40 VA radiation oncology practices were abstracted from the electronic medical records, treatment management, and planning systems as part of the pilot. The pilot demonstrated that clinical measures provide a tangible means to quantify and improve quality of care.^4^ It also proved that manual data abstraction is time‐consuming, onerous, and very expensive. It clearly established the need for IT infrastructures for automatic data abstraction and curation.^5^

There have been several IT initiatives by research groups in radiation oncology to develop integrated data analysis platforms for either outcome studies and/or decision support systems.^6^ There are many large databases such as Surveillance, Epidemiology and End Results (SEER) program established by the National Cancer Institute (NCI) in 1973 and Center of Medicare and Medicaid Services (CMS) that collect data from large number of cancer patients treated over time.^7^ The data in these databases include demographics, cancer incidence, clinical, and survival factors but fail to include detailed clinical and treatment information. Some of the data analyses from the SEER database suggests that the database lacks information about the radiation dose, technique, and radiotherapy receipt.^8^

The University of Michigan has developed M‐ROAR^9^ and MROQC^10^ as data aggregation systems to collect and assess practice patterns, perform outcome analyses, and evaluate dosimetry‐related information. MD Anderson has implemented a system‐wide electronic data capture system that records patients’ treatment information.^11^ Johns Hopkins has launched the Oncospace program that captures RT data containing anatomy, dose distributions, and outcomes data in an analytical database.^12^ The Mayo Clinic’s Department of Radiation Oncology in Florida has linked its radiation oncology information system with Mayo Clinic’s internal claims data warehouse along with Mayo’s tumor registry which allows for large‐scale studies.^13^ Whitaker et al. at Mayo Clinic at Rochester developed a patient‐reported outcome (PRO) collection and management system to implement a large‐scale aggregation of patients’ treatment data. A majority of these platforms are deployed with very specific goals. Whereas, the HINGE software is designed to collate more comprehensive radiotherapy episodic data that include DICOM‐RT data from TPS, treatment data from TMS, and clinical data from EHR. The overarching goal of HINGE is to meet the following objectives:

1. To allow health care institutes to assess their practices/treatment outcomes and make improvements at a systemic level;
2. To better equip and assist the physician with complimentary/supplementary information to aid their clinical decision‐making process;
3. To create systems that would allow for the research and development of tools that relate to machine learning, artificial intelligence, and big data analytics;
4. To allow for ease of data interoperability, data access, and exchange for third‐party applications/programs;
5. To foresee the future trends in the healthcare industry and subsequently design data platforms in alignment with the upcoming technologies.

## 3 OVERVIEW OF THE HINGE PLATFORM

**Fig. 2:**
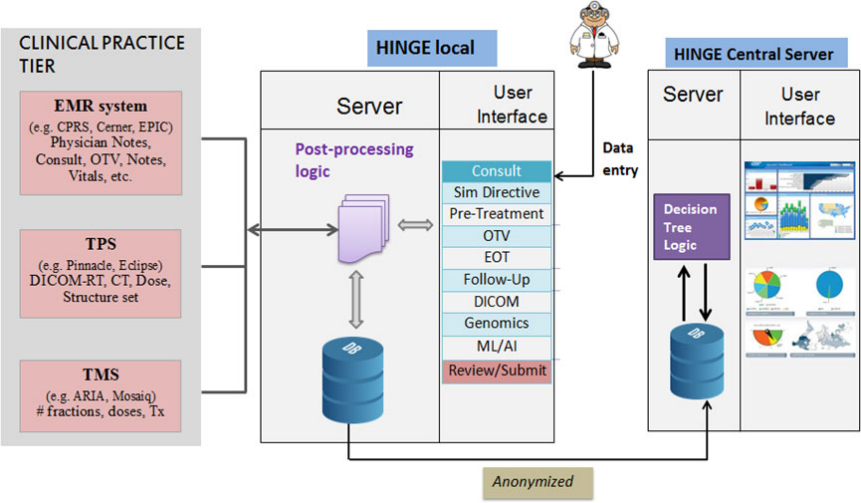
Overview of the architecture of Health Information Gateway and Exchange (HINGE) software platform: The clinical workflow templates (Consult, Sim Directive, etc.) in the HINGE local are automatically populated with data that are available in clinical practice systems that include electronic health record (EHR), treatment planning system (TPS), and treatment management system (TMS). The complete radiotherapy data are sent to the HINGE central server, where it is evaluated for data integrity, curated, and prepared for visualization by end users in a web‐based graphical user interface (GUI).

The crucial data elements required to assess the quality of radiotherapy planning and delivery and to build decision support systems are distributed across disparate clinical systems and are recorded along each sequential step of the radiation treatment (from initial Consult to follow‐up). Figure 2 shows a brief overview of the HINGE architecture. HINGE is a real‐time data analytics portal connecting the EHR, TPS, and TMS. The HINGE local application resides at each local facility, and the HINGE Central Server is hosted at a HIPPA compliant secure cloud server. Radiation oncologists enter the information via smart disease‐specific templates (user interface), which is discretized and stored in the database. HINGE Local also communicates with EHR, TPS, and TMS and imports/exports relevant patient data. The data are anonymized and sent to HINGE Central Server for data analytics and display of results on an interactive Web‐based dashboard for end‐users.

## 4 KEY DESIGN FEATURES

### 4.A Data standardization

**Fig. 3:**
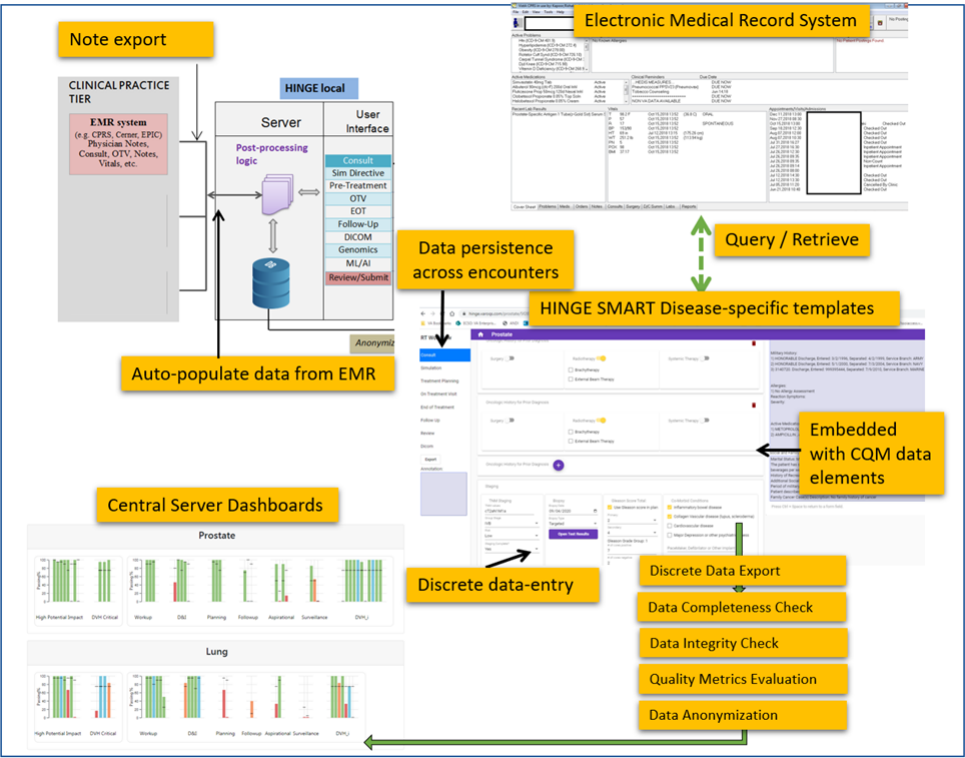
Overview of the components of the Health Information Gateway and Exchange (HINGE) application: Discrete clinical data abstracted via query/retrieve from the Electronic medical record (EHR) and populated in the HINGE SMART disease‐specific templates UI. Discrete and free‐text data are transcribed by the providers in the disease‐specific templates. SMART templates have business logic to auto calculate scores, perform auto‐population of subsequent templates with discrete data, report any missing value or value outside a defined range, and abstract the data elements for clinical quality measure (CQM) analysis. A free‐text narrative note is generated from all these discrete data elements and interfaced to the EHR as part of the clinical documentation. All the data from these SMART templates are checked for completeness and integrity and anonymized before exporting it to the Central Server Dashboard where data visualization tools (charts, graphs with flagging of outliers, etc.) are deployed to analyze the CQMs, clinical and dosimetry data for a cohort of patients.

HINGE software is designed to facilitate the process of extract, transform, and load (ETL) of data throughout. Disparate systems are used to collect clinical, treatment, and process data; however, the lack of uniformity in data syntax and semantics makes it extremely difficult for data aggregation and analysis. Unfortunately, at the present time, there is a paucity of ontologies with radiation oncology‐specific terms. HINGE deploys “smart” disease‐specific templates meant for data entry/viewing as part of its user interface for radiation oncologists. These templates facilitate the physicians and the clinical staff to enter the relevant clinical information in a discrete manner. Figure 3 shows the overview of the components of the HINGE application. The templates are embedded with critical data elements (data farming) that are required for QA analyses. These critical data elements are used to score the disease site‐specific CQMs that are listed in the paper from Hagan et al.^4^ Most commonly, electronic case report form templates are utilized routinely to collect structured data in randomized controlled trials, but these templates are limited to trial‐specific data elements, and those are entered in addition to routine clinical documentation. These templates are utilized as part of the routine clinical workflow and documentation and are the starting point for the physicians to record their assessments. The templates mimic the radiotherapy workflow from consultation, simulation, treatment, and end‐of‐treatment to follow‐up care. The templates are interfaced with the EHR, allowing data such as allergies, drug list, lab values, and vitals to be automatically populated into the template from the EHR database. Thus, the templates facilitate the entry of data in a structured discrete format, along with simultaneously allowing free‐text data entry sections for recording additional observations. However, much of the data such as TNM staging, performance status, treatment intent, status, previous cancer encounters with RT, chemotherapy or surgery as the treatment modality, prescription, toxicity grades, simulation, treatment planning directive, and survivorship data elements are entered in discrete format. We used all predefined radiotherapy data nomenclature (AJCC,^14^ CTCAE,^15^, ^16^ and AAPM TG 263^17^) and defined additional ones where no standard data definitions existed. Automatic calculation of assessment scores and graphical indication of treatment progress are rendered in these templates. At each encounter, after the data entry has concluded, HINGE prepares the data into a textual narrative note format by utilizing user specified template boilerplate narratives and embedding these discrete data elements. The full note narratives are then exported to the EHR for medical records via an interface for the purpose of maintaining clinical documentation and continuity of care because the patient might be subsequently seen at other clinical services within the hospital. These templates have been developed with specific UI‐based design considerations from the physicians' end users. These templates are specifically designed to save physicians’ time/effort and enhance their ease of access by incorporating technical UI/UX features like least amount of page scrolling, reducing the number of mouse clicks, data entry in a lateral motion within the HINGE application, positioning high‐utility patient details on the top of the page, business logic for auto calculation of certain data elements such as NCCN risk groups based on staging, and Gleason and PSA values. In addition, auto population of subsequent note templates (e.g., end of treatment template) with discrete data from previous templates (consult, treatment planning directive template) also saves physician time that they can spend with the patients rather than just dictating notes in the EHR. The templates also perform extensive data entry validation and data‐completeness check at the entry level and provide helpful error messages, suggestions, highlighting of critical elements, and so on. These templates are disease site specific, and relevant data entry fields appear based on the diagnosis and treatment site codes. The templates also prepopulate the data fields from TPS and TMS so that the physicians do not have to make redundant entries.

### 4.B Integration with radiotherapy data sources

#### 4.B.1 EHR‐HINGE integration

**Fig. 4:**
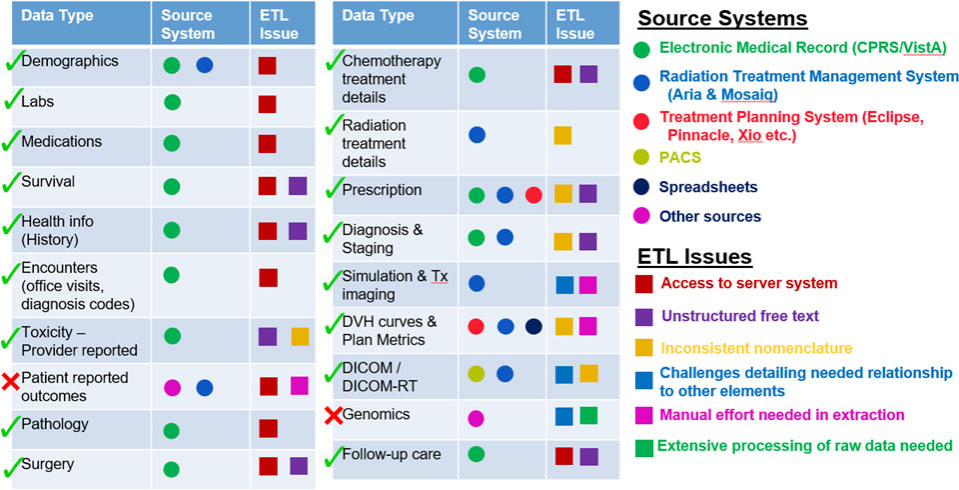
List of the data types utilized in radiation oncology domain, source system where the data resides, extract/transfer/load (ETL) issues. Access to server system, unstructured free‐text, and inconsistent nomenclature are among the major ETL issues across the various source systems. Health Information Gateway and Exchange (HINGE) application gathers data types (green tick) from almost all the mentioned source systems.

HINGE is designed to communicate with VHA’s EHR, that is, VISTA. HINGE has employed an external interface that is able to communicate (query/retrieve) with VISTA and fetch required patient details such as demographics, vitals, labs, medications, surgery, pathology, encounter, allergies, and survival information. The list of data types retrieved from the EHR is shown in the Fig. 4. Most of the information exists in discrete format when it is retrieved from the EHR. The interface is also able to retrieve information such as health history, surgery, and radiology reports that only exist as clinical free‐text notes from the EHR. Additionally, after the note is completed by the physician, the discrete data are converted into a textual note and exported to VISTA via this interface. Specifically, this interface‐based design allows HINGE to be oblivious to the underlying EHR system (VISTA, Cerner, EPIC, etc.). It helps in its portability and allows it to be functional even if the EHR system changes by isolating the business logic of the integration strictly within the interface.

#### 4.B.2 TPS‐HINGE integration

**Fig. 5:**
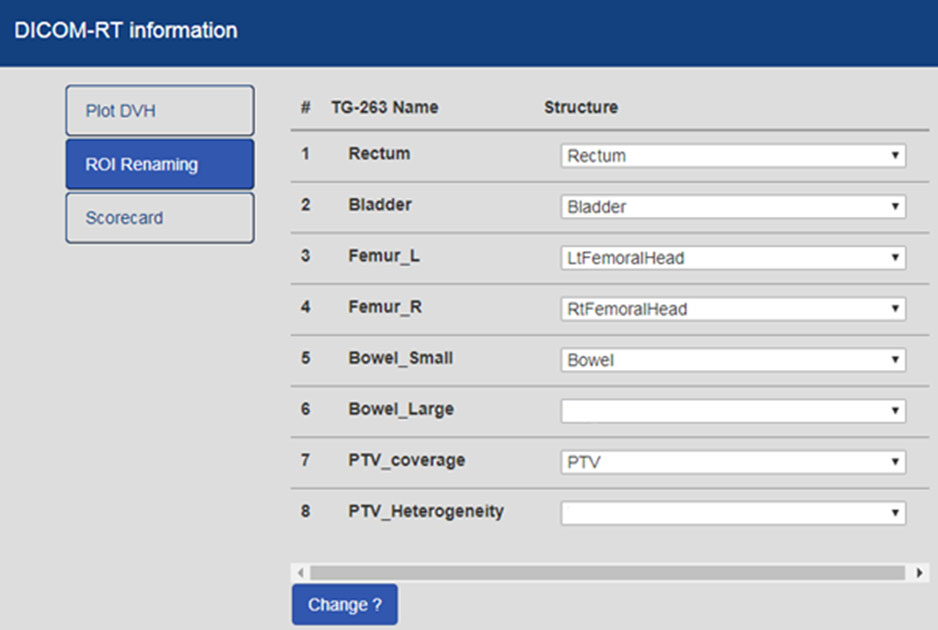
Screen capture of the user interface for selecting the appropriate structures for target and organ at risk (OAR) renaming in the Health Information Gateway and Exchange (HINGE) application.

HINGE is able to import DICOM‐RT data from any TPS that conform to the Integrating the Healthcare Enterprise–Radiation Oncology (IHE‐RO)^18^ defined profiles. The VA system utilizes all the TPS products sold in the marketplace, and hence, it is imperative that we conform to one standard interoperability solution provided by IHE‐RO to pull data for an enterprise‐wide application such as HINGE. One of the major challenges with examining patients’ DICOM‐RT data is the lack of standardized target and organ at risk (OAR) nomenclatures, prescription formatting, and ambiguity regarding dose‐volume histogram metrics, across several disease sites. This impedes any research into examining dosimetric effects of practice patterns longitudinally. To resolve this issue, an initiative to introduce the standardizing nomenclature for radiotherapy was implemented under TG‐263.^17^ HINGE deploys this naming convention within its system, requiring treatment planners to match the deemed OARs to their TG‐263 names. HINGE automatically suggests the equivalent TG‐263 names for the listed OARs for the planner (Fig. 5). In addition to simple text mapping, Machine Learning based methods are being used to automate the process of relabeling physician specified target and OAR names to the TG‐263 specified names. Success in this approach has been shown using target and OAR text labels,^19^ geometric information,^20^, ^21^, ^22^ and radiomics features,^23^ all found in the DICOM structure set, dose, and reference imaging (CT) datasets. All these methods have shown reasonably good accuracy over many different structure types, and the HINGE platform has the capability of deploying such methods as it has all of the treatment planning DICOM files as well as access to cloud‐based machine learning frameworks including Amazon Web Services (AWS) Elastic Map Reduce and Deep Learning Containers. The software calculates and displays DVH from the uploaded dataset to the dosimetrist for final verification and selection of the key target and OAR sites. Based on the DVH dose constraint‐based quality measures, the appropriate pass/fail/acceptable variation status is stored in the database before the complete dataset is uploaded to the central server dashboard.

#### 4.B.3 TMS‐HINGE integration

Existing TMSs are primarily designed to optimize the clinical workflow and therefore lack utilities required to facilitate big data applications. For HINGE to assume the role as the one‐stop‐shop for managing radiotherapy data, it must have access to the data present in TMSs such as Varian Aria/Elekta Mosaiq products. To achieve this goal, we have created a Docker container, named HINGE‐Broker, which runs on the same network as the TMS’s underlying database. Within HINGE‐Broker, a Python script provides access to the TMS database using the SQLAlchemy toolkit, and a Node application exposes a web API for HINGE to make remote queries. The TMS database contains discrete elements such as patient demographics, prescribed prescriptions, delivered dose, and a listing of all clinical notes.

However, TMS software upgrades may result in changes to the underlying database schema, and potentially different versions of TMS deployed across a health care system will also require the support of multiple methods of data access. In addition, some versions of TMS require very complicated, nonintuitive SQL queries for retrieving current dose information, which potentially makes this approach very sensitive to schema changes. Although the TMS software provides methods for directly entering many discrete data elements, these tools are often underutilized, resulting in little information that could be used for further studies. The data extracted from TMS are used to populate the simulation, undertreatment, and end of treatment summary templates in HINGE and are made available for physicians to view/edit. This allows for HINGE to access treatment delivery data in a discretized manner.

Radiotherapy TMS does support the storage and display of Word documents within the application. We have partially addressed the lack of discrete treatment data by creating Word templates for the undertreatment visit and end of treatment summary notes with tagged fields. Using a Word macro, these discrete template fields can be exacted as JSON, which can then be stored in a MongoDB database for analysis.

### 4.C QA/analyses of radiotherapy data

**Fig. 6:**
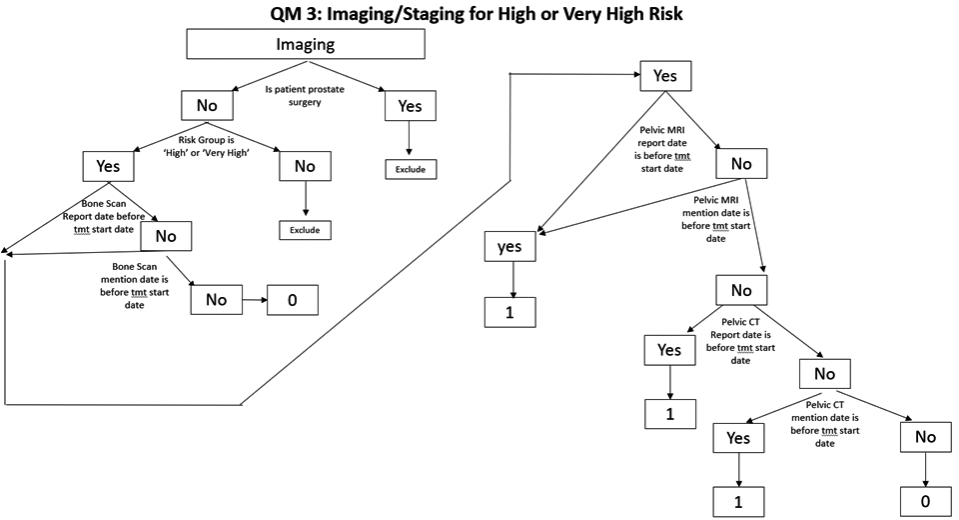
Example of a decision tree logic for a clinical quality measure. Data from the health information gateway and exchange (HINGE) SMART templates, treatment planning system (TPS), and treatment management system (TMS) modules are utilized with these decision trees to generate pass/fail (1/0) for each of the disease site‐specific clinical quality measures.

With data standardization and clinical integration described in the preceding sections, HINGE software is able to aggregate the critical data elements from the entire clinical workflow to score all CQMs for each patient. Once a patient’s treatment is deemed complete for export, the anonymized data are uploaded to the HINGE’s central server. The business logic for deriving the CQMs based on the patient data resides on the central server (see Fig. 2). After the data are received, the CQMs are calculated and are available for viewing on the visual dashboards on the central server via a web portal. Many of the CQMs are not straightforward and require extensive decision‐tree logic to construe a “pass” or a “fail.” HINGE has deployed such decision‐tree logic (Fig. 6) in its system to calculate the CQM scores automatically for each CQM. The automated real‐time calculation and evaluation of treatment data allow the physicians to benchmark their treatment practice against their peers across the VA enterprise, thereby providing a real‐time feedback. Additionally, postcompletion of treatment, HINGE is able to generate a treatment scorecard that evaluates each patient’s RT treatment. This allows the VHA program office and quality managers to assess the quality of RT in the VA clinics.

### 4.D Dashboard analytics

**Fig. 7:**
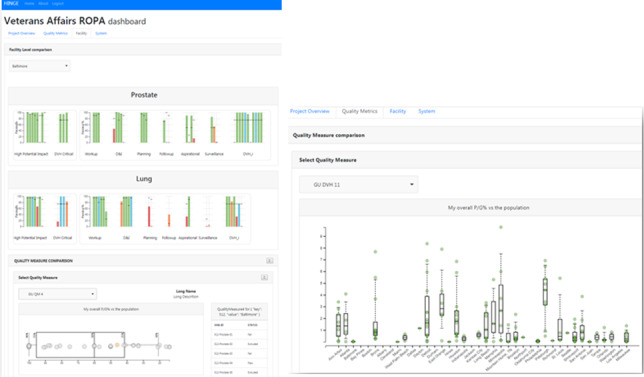
Screen capture of the health information gateway and exchange (HINGE) dashboard application showing data from 40 VA practices.

HINGE visual dashboards available through the central server display visual plots, charts, and graphs each detailing the performance of every VA practice for each CQM (Fig. 7). The dashboard provides a vantage position for every physician and the quality managers to assess the performance of each CQM relating to its current standing, in comparison with expert‐defined thresholds and their peers’ performance nationwide. This allows the physician to understand the quality of RT care they deliver and ways to improve it. The dashboard also provides the quality managers with insightful information to investigate performance or systemic issues, marshal resources, and design effective health policy solutions to improve RT care.

In addition to viewing the information through the dashboard, the central server functions as the data warehouse because all the patient data from local VA facilities are exported to this data warehouse. Thus, it is poised for large enterprise analytics that involve data mining, outcomes research, comparative effectiveness, machine learning, and other large database interrogation queries.

### 4.E Data anonymization

By way of architectural design, HINGE is split into HINGE local and HINGE central server (Fig. 2). HINGE local is the application facing the local VHA facility connected to the local environment and resources such as EHR, TPS, and TMS. The HINGE local application is hosted on the cloud computing platform hosted by VA’s Enterprise cloud (VA‐EC)–AWS, and the application is running multiple instances with the database siloed into partitions for each local VA center. HINGE central server is hosted on a VA‐EC as well where data are captured from each of the HINGE‐local instances. After a treatment is completed, the physician/clinical staff are prompted to review the patient data and send it to the central server. After the clinical staff at a local facility initiate the export of data, the data are anonymized and presented for review and approval. In this review, the protected health information is removed from the treatment data collected from all clinical and treatment management notes, DICOM‐RT datasets is presented to the attending physician for their final approval. The de‐identified data are compliant as per HIPAA policies. After approval, the data are exported from HINGE local to the central server via a secure encrypted channel.

### 4.F Data security

Data security is paramount and a crucial component of any health care organization and infrastructure. The numbers of cyber‐attacks on the health care industry are constantly growing for the purposes of medical identity theft and Medicare fraud. HIPPA regulations^24^ set specific guidelines for maintaining the privacy and security of any information system deployed in the health care domain. The HIPPA security rules outline the administrative, physical, and technical security measures that an organization must take to ensure confidentiality, integrity, and availability of health care datasets.^25^, ^26^ HINGE is utilizing administrative safeguards where documented policy and procedures are established to create a uniform process that clinical users follow to maintain patient privacy and information security in the software. HINGE also employs technical safeguards where no PHI/PII is shared within or with other interfaced application without appropriate network (SSL) and software encryption. The VHA has very stringent physical safeguards in place where the data centers housing the HINGE application have locks and security system to protect from PHI data breaches associated with break‐ins. Keeping the health care data confidential, available and maintaining integrity have direct relationships with HIPAA compliance.

Confidentiality is the act of ensuring that patient’s health data are kept completely undisclosed to unauthorized entities. HINGE is integrated with the VA’s single sign on (SSO) and 2‐factor authentication (2FA, token key and password) system where enterprise wide access control measures are undertaken by the VA’s central IT office. Having the HINGE software run on the cloud environment leads to an increase in the risk of data compromises, as the data become accessible to an augmented number of subsystems. In the HINGE software design architecture, we have made the application tools self‐contained thereby mitigating the risk that comes with connecting with third party vendor tools. The electronic interfaces with the EHR and treatment management & planning systems are also unidirectional with the intention to pull the data utilizing proper software encryption modules.

Integrity is important to make sure that the health care data captured by HINGE are accurate and consistent and not modified in any way. Treatment decisions based on erroneous data can have serious and adverse consequences on patients' health. HINGE utilizes checksum or a hash, before using the data, and if integrity check fails, the application reports an error in an audit trail and terminates the transaction without processing the data.

For the HINGE application to be successful and serve its purpose, the information must be available at all time in spite of service disruptions due to hardware failure, system upgrades, power outages, and denial of service attacks. The deployment of the application is on two separate AWS availability zones with load balancers and multiple redundant copies of the MongoDB backend database to ensure high availability.

## 5 DISCUSSION

Collation of comprehensive population‐based clinical information, radiation treatment planning, delivery, and health outcome information is essential for any robust radiation oncology quality surveillance and outcome assessment program. The HINGE software platform allows passive real‐time assessment of a radiotherapy quality of care. The quality managers can now grade every treatment against established rubric of nationwide norms of outcome, toxicity, and treatment delivery. Some additional benefits include reducing the burden of basic data collection for quality analysis and creating a single‐point capture of source data, which protects data integrity by eliminating manual transcription of data from multiple sources and provides better data traceability and provenance, while reducing the need for data queries, data cleaning, and source data verification—processes that within themselves hold the potential for errors. Furthermore, data collected in the HINGE can be used to create decision support models in the clinical systems that enable clinicians to improve the quality and safety of care rendered to the patients. With the increasing emphasis on delivery of value‐based health care, the HINGE system can not only quantify value and quality of care but also aggregate outcome data using standard templates/data elements. An alternative approach to collecting data for quality surveillance and outcome research is to leverage NLP for the extraction of discrete data from unstructured clinical documentation. However, there are several challenges with this approach. The free‐text clinical notes have many different taxonomies, vocabularies, terms, or abbreviations that are often used by clinicians because there are currently no standards that are universally adopted in radiation oncology domain. The lack of standardization of information presented in the free‐text notes makes traditional NLP solutions difficult to implement. Syed et al.^27^ recently reported on an integrated machine learning/NLP model using the fast text algorithm^28^ for standardizing the OAR names in the DICOM RT structure set files with the TG‐263 specified standard names. The results for prostate and lung datasets reported high *F*_1_ scores on OAR names but low scores on tumor/target names due to a wide variability of nonstandard names utilized for targets. In many cases, even when presented a consistent vocabulary/taxonomy, it is challenging for the NLP algorithm to decern information because much of the clinical meaning in free‐text blobs is context based, and it requires specific decision tree logics with multiple expression values to extract a single data element.

Another key feature of the next release of software will be the integration of PROs. We plan to deploy an infrastructure with public patient facing web‐based tools to capture longitudinal PRO data within HINGE to facilitate earlier interventions, rapid symptom management, and track patient reported quality of life assessments. A similar public facing web‐based tool will be deployed to collect radiotherapy treatment data from community radiation oncology providers that are currently treating over 60% of veteran cancer patients. Such a strategy will allow us to aggregate radiotherapy data for over 45 000 veterans treated annually in the community and at 41 VHA sites. This has the potential for big data outcome research in radiation oncology and high‐quality continuity of care. Finally, the development of future versions of HINGE software will be coordinated with the medical and surgical oncology programs to ensure harmonization of clinical workflow templates among all cancer care specialities.

For big data and smart health care techniques to succeed in medicine, it is imperative that all stakeholders—physicians, physicists, nurses, clerks, and commercial vendors—work together on how and what data needs to be collected. The funding agencies such as National Institute of Health and NCI should direct their resources to support the work around integrating the clinical practice with automating and streamlining clinical workflow around structured data collection methodologies and define clinically meaningful measures of care rendered to our patients. The process used to create the HINGE database and model can be replicated for all domains of medicine where each domain is responsible to define their own workflow templates, clinical measures, and data analysis tools that can be used as a feedback to the practice for quality improvement.

## CONFLICT OF INTEREST

No conflicts of interest to report from the author team.

## AUTHOR CONTRIBUTIONS

All the authors listed above have made substantial contributions in the design, build, analysis, and implementation of the system mentioned in the manuscript. This work has been jointly carried out from the team from the US Veterans Healthcare Administration and Virginia Commonwealth University. All the authors have made significant contributions in drafting and critically reviewing the manuscript text and figures. All the authors have provided their approval with the final version of the submitted manuscript.

## References

[1] Spasić I , Livsey J , Keane JA , Nenadić G . Text mining of cancer‐related information: review of current status and future directions. Int J Med Inform. 2014;83:605–623. 10.1016/j.ijmedinf.2014.06.009. Epub 2014 Jun 24 PMID: 25008281. 25008281

[2] Fleishon HB , Wald C , Korn R , Rosenthal S , Fredericks N . The clinical research center: a vital part of the ACR mission. J Am Coll Radiol. 2011;8:422–427 PMID:21636057. 21636057 10.1016/j.jacr.2010.12.015

[3] Crozier C , Erickson‐Wittmann B , Movsas B , Owen J , Khalid N , Wilson JF . Shifting the focus to practice quality improvement in radiation oncology. J Healthc Qual. 2011;33:49–57. PMID:23845133. 10.1111/j.1945-1474.2011.00119.x 23845133

[4] Hagan M , Kapoor R , Michalski J , et al. VA‐radiation oncology quality surveillance program. Int J Radiat Oncol Biol Phys. 2020;106:639–647. 31983560 10.1016/j.ijrobp.2019.08.064

[5] Caruthers D , Brame S , Palta JR , et al. Development and implementation of quality measures for the survey based performance assessment of radiation therapy in the VA. Int J Radiat Oncol. 2017;99:E391–E392.

[6] Matuszak MM , Fuller CD , Yock TI , et al. Performance/outcomes data and physician process challenges for practical big data efforts in radiation oncology. Med Phys. 2018;45:e811–e819. 30229946 10.1002/mp.13136 PMC6679351

[7] Seer.cancer.gov . Surveillance, Epidemiology, and End Results Program. 2017. Available at http://seer.cancer.gov/.

[8] Jagsi R , Abrahamse P , Hawley S , Graff J , Hamilton A , Katz S . Underascertainment of radiotherapy receipt in Surveillance, Epidemiology, and End Results registry data. Cancer. 2011;118:333–341. 21717446 10.1002/cncr.26295 PMC3224683

[9] Mayo CS , Kessler ML , Eisbruch A , et al. The big data effort in radiation oncology: data mining or data farming? Adv Radiat Oncol. 2016;1:260–271. 28740896 10.1016/j.adro.2016.10.001 PMC5514231

[10] Moran JM , Feng M , Benedetti LA , et al. Development of a model web‐based system to support a statewide quality consortium in radiation oncology. Pract Radiat Oncol. 2017;7:e205–e213. 28196607 10.1016/j.prro.2016.10.002

[11] Pasalic D , Reddy JP , Edwards T , Pan HY , Smith BD . Implementing an electronic data capture system to improve clinical workflow in a large academic radiation oncology practice. JCO Clin. Cancer Inform. 2018;1–12. 10.1200/CCI.18.00034 PMC6874007 30652599

[12] McNutt TR , Evans K , Wu B , et al Oncospace: all patients on trial for analysis of outcomes, toxicities, and IMRT plan quality. Int J Radiat Oncol Biol Phys. 2010;78:S486.

[13] Waddle MR , Kaleem T , Niazi SK , et al Cost of acute and follow‐up care in patients with pre‐existing psychiatric diagnoses undergoing radiation therapy. Int J Radiat Oncol. 2017;99:1321. 10.1016/j.ijrobp.2019.03.021 30904707

[14] AJCC . American Joint Committee on Cancer. 2017. Available at. https://cancerstaging.org/Pages/default.aspx.

[15] Ctep.cancer.gov . Common Terminology Criteria for Adverse Events (CTCAE). 2017. Available at: https://Ctep.cancer.gov/protocoldevelopment/electronic_applications/ctc.htm [Accessed 15 Nov. 2017].

[16] Basch E , Pugh SL , Dueck AC , et al. Feasibility of patient reporting of symptomatic adverse events via the patient‐reported outcomes version of the common terminology criteria for adverse events (PRO‐CTCAE) in a chemoradiotherapy cooperative group multicenter clinical trial. Int J Radiat Oncol Biol Phys. 2017;98:409–418. 28463161 10.1016/j.ijrobp.2017.02.002 PMC5557037

[17] Mayo C , Moran JM , Xiao Y , et al AAPM Task Group 263: tackling standardization of nomenclature for radiation therapy. Int J Radiat Oncol. 2015;93:E383–E384.

[18] Rengan R , Curran B , Able C , et al. Addressing connectivity issues: the integrating the healthcare enterprise‐radiation oncology (IHE‐RO) initiative. Pract Radiat Oncol. 2011;1:226–231. 24674000 10.1016/j.prro.2011.06.016

[19] Syed K , Sleeman W , Ivey K , et al. Integrated natural language processing and machine learning models for standardizing radiotherapy structure names. Healthcare. 2020;8:120 10.3390/healthcare8020120 PMC7348919 32365973

[20] Rhee D . TG263‐Net: A deep learning model for organs‐at‐risk nomenclature standardization. Med Phys. 2019:46. No. 6. 111 RIVER ST, HOBOKEN 07030‐5774, NJ USA: WILEY.

[21] Yang Q . A novel deep learning framework for standardizing the label of OARs in CT. Workshop on Artificial Intelligence in Radiation Therapy. Cham: Springer; 2019.

[22] Sleeman IV WC , Nalluri J , Syed K , et al. A Machine learning method for relabeling arbitrary DICOM structure sets to TG‐263 defined labels. J Biomed Inform. 2020;109:103527. 32777484 10.1016/j.jbi.2020.103527

[23] Sleeman C . Relabeling Non‐Standard to Standard Structure Names Using Geometric and Radiomic Information. Med Phys. 2020:47. No. 6. 111 RIVER ST, HOBOKEN 07030‐5774, NJ USA: WILEY.

[24] Accountability Act . Health insurance portability and accountability act of 1996. Publ Law. 1996;104:191.

[25] Metri P , Sarote G . Privacy issues and challenges in cloud computing. Int J Adv Eng Technol. 2011;5:5–6.

[26] Andress J . The Basics of Information Security: Understanding the Fundamentals of InfoSec in Theory and Practice. Boston, MA, USA: Syngress; 2014.

[27] Syed K , Sleeman W IV , Ivey K , Hagan M , Palta J , Kapoor R , Ghosh P . Integrated natural language processing and machine learning models for standardizing radiotherapy structure names. Healthcare. 2020;8:120. Multidisciplinary Digital Publishing Institute. 10.3390/healthcare8020120 PMC7348919 32365973

[28] Wu S , Manber U . Fast text searching: allowing errors. Commun ACM. 1992;35:83–91.

